# Diagnosis of Alzheimer's Disease Using Dual-Tree Complex Wavelet Transform, PCA, and Feed-Forward Neural Network

**DOI:** 10.1155/2017/9060124

**Published:** 2017-06-21

**Authors:** Debesh Jha, Ji-In Kim, Goo-Rak Kwon

**Affiliations:** Department of Information and Communication Engineering, Chosun University, 309 Pilmun-Daero, Dong-Gu, Gwangju 61452, Republic of Korea

## Abstract

*Background*. Error-free diagnosis of Alzheimer’s disease (AD) from healthy control (HC) patients at an early stage of the disease is a major concern, because information about the condition’s severity and developmental risks present allows AD sufferer to take precautionary measures before irreversible brain damage occurs. Recently, there has been great interest in computer-aided diagnosis in magnetic resonance image (MRI) classification. However, distinguishing between Alzheimer’s brain data and healthy brain data in older adults (age > 60) is challenging because of their highly similar brain patterns and image intensities. Recently, cutting-edge feature extraction technologies have found extensive application in numerous fields, including medical image analysis. Here, we propose a dual-tree complex wavelet transform (DTCWT) for extracting features from an image. The dimensionality of feature vector is reduced by using principal component analysis (PCA). The reduced feature vector is sent to feed-forward neural network (FNN) to distinguish AD and HC from the input MR images. These proposed and implemented pipelines, which demonstrate improvements in classification output when compared to that of recent studies, resulted in high and reproducible accuracy rates of 90.06 ± 0.01% with a sensitivity of 92.00 ± 0.04%, a specificity of 87.78 ± 0.04%, and a precision of 89.6 ± 0.03% with 10-fold cross-validation.

## 1. Introduction

Alzheimer's disease (AD) is an irremediable neurodegenerative disorder that causes dementia in elderly people around the globe. It has been predicted that the pervasiveness of AD will double within the next 2 decades and that one out of every 85 people will be afflicted with the disease by 2050 [1]. Therefore, there is a need to identify neuroimaging biomarkers that can grant accurate and early diagnoses of dementia. In addition, to diagnose an AD sufferer clinically at a primitive disease stage, many imaging biomarkers must be identified using different imaging modalities, such as MRI [2], position emission tomography (PET) [3], functional magnetic resonance imaging (fMRI) [4], single-photon emission computed tomography (SPECT) [5], and magnetic resonance spectral imaging (MRSI) [6].

An accurate and early diagnosis of AD and identification of the risk of progression from mild cognitive impairment (MCI) to AD provide AD sufferers with awareness of the condition's severity and allow them to take preventative measures, such as making lifestyle changes and taking medications [7]. Currently, many neurologists and medical analysts have been spending significant time to researching technique to allow for early diagnosis of AD, and encouraging results have been frequently achieved [8]. MRI is an influential, noninvasive brain imaging technique that provides higher-quality information about the shape and volume of the brain than computed tomography (CT), SPECT, and PET scans. It provides superior soft tissue differentiation, high spatial resolution, and better contrast and can even identify tiny irregularities in the brain [9]. Moreover, the diagnostic use of MRI has been tremendously improved due to the automated and precise labeling of MR images, which performs an important role in identifying AD in related patients from healthy and elderly controls (HC) [10].

Earlier, majority of diagnosis work was accomplished manually or semimanually for measuring a priori region of interest (ROI) of MRI, based on the reality that subjects with AD experience have more cerebral atrophy when compared to HCs [11, 12]. Most of this ROI-based examination focused on the contracting of the cortex and hippocampus and amplified ventricles. Nevertheless, ROI-based approaches are not practicable in hospitals because of few shortcomings: (i) ROI technique needs a priori data and expert knowledge. (ii) The manual diagnosis accuracy is dependent on the knowledge of physicians and interpreter [13]. (iii) The interaction among the voxels was troublesome to enforce. (iv) It was essential to explore other potential areas that may be linked to AD [14]. (v) Automatic segmentation of ROI was not beneficial in practice, and investigator needed to segment the brain using hand [15]. Therefore, automated methods can assist physician in diagnosing diseases from images such as those produced by MRI, for which many slices are extracted from the tissues and long periods of may be necessary for the evaluation of the images.

The aim of this article is to present an automated approach for diagnosing AD by using the “whole brain analysis” method. It has achieved popularity, since it examines entire voxels of the brain. It is not essential to segment the brain as earlier, and it does not require any biomarker for the classification purpose. The main drawback is dimensionality that can be resolved through high-speed computers, which is comparably inexpensive [16]. The whole-brain investigation laboriously relies on true computation, and it can only be finished by a computer researcher after a physician assisted in labeling the input data as either AD or HC. Usually, the whole-brain inspection labels the entire brain as a ROI, where two stages are involved, namely, feature extraction and classification.

Scholars have presented different methods to extract effective features for the detection of AD and other types of pathological brain disease. Additionally, classification models and methods survive; nevertheless, not all of them are suitable for the processing of MR brain images. Based on latest literature, we found two drawbacks with the previous work: (i) The discrete wavelet transform (DWT) is usually utilized for feature extraction. The DWT has better directional selectivity in horizontal, vertical, and diagonal directions and has better image representation than Fourier transform, but its major drawbacks are that it has poor directionality, is sensitive to shifts, and lacks phase information. (ii) Most of the state-of-the-art mechanisms consider only single slice-based detection (SSD) per patient. The obtained slices may not contain the foci of the disease.

To tackle above problems, we suggested two improvements. First, we propose a DTCWT that possesses attractive properties for image processing, including shift invariance and high directionality. Second, we consider multiple slices for each patient unlike previous studies, so that information gain is more consistent, reliable, and accurate. In hospitals, multiple slice-based detection is utilized because of its inexpensiveness. Research has clearly showed that the DTCWT is more suitable than the traditional wavelet domain for feature extraction [17].

Our contribution aims to introduce a novel method for AD detection with higher accuracy than state-of-the-art methods, on the basis of DTCWT, PCA, and ANN technique. Furthermore, we build a computer-aided diagnosis (CAD) system, which can be utilized in the early diagnosis of AD-related brain area and subjects. Our objective is to develop assisting tool for clinicians.

All of the preprocessing methods are used to obtain good results. To show effectiveness of our proposed system, we have evaluated performance measures including accuracy, sensitivity, specificity, precision, and bar plot for the comparison of the proposed method with the existing systems. The paper is arranged as follows.  Section 2 offers background knowledge on materials and methods. In Section 3, the experiments, results, and discussion are presented. Finally, conclusion and plan for future studies are presented in Section 4.

## 2. Materials and Methods

### 2.1. Materials

#### 2.1.1. Dataset

**Figure 1:**
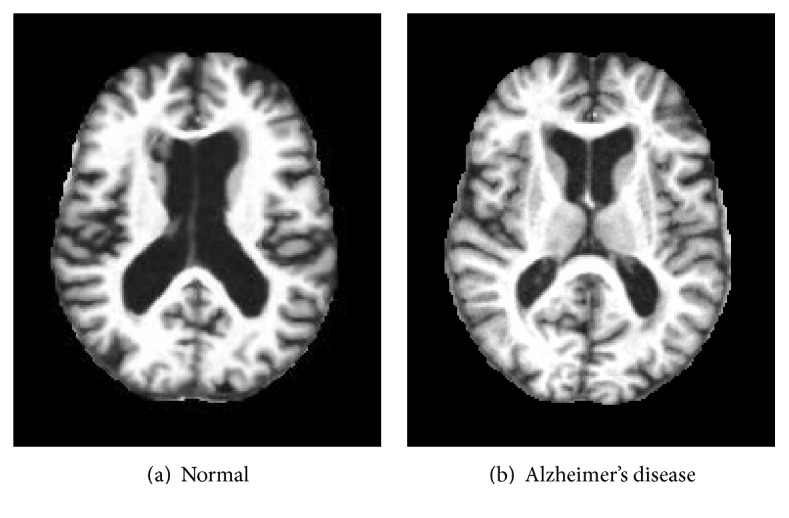
Dataset sample (axial view after preprocessing).

In our study, the dataset is accessed from Open Access Series of Imaging Studies (OASIS). OASIS is a project, for compiling and sharing MRI datasets of the brain to make such data accessible to the scientific community. The data are accessible at http://www.oasis-brains.org. A sample of the MR brain image is shown in Figure 1.

OASIS provides two types of data: cross-sectional and longitudinal MRI data. In this study, we used cross-sectional MRI data because we aimed to develop an automatic system for detecting AD, which would not require longitudinal data that had been gathered from AD patients over long periods of time.

The dataset consists of 416 subjects whose ages are between 18 and 96. In our study, we consider 126 samples (including 28 ADs and 98 HCs).  Table 1 shows statistical information about the subjects included in the experiment. Only right-handed subjects are included in the study, consisting of both men and women. The exclusion criterion is patients less than 60 years of age or any of their reports are missing. The unbalanced data may cause difficulty in future recognition; we fine-tune the cost matrix to resolve this issue.

The dataset contains information about the patient's demographics. The demographic features contain gender (M/F), age, education, socioeconomic status, and handedness. The mini mental state examination (MMSE) is a short 30-point questionnaire test utilized to monitor for cognitive impairment and dementia. The MMSE test comprises simple questions and problems in numeral areas: the time and place, repeating list of words, arithmetic, language utilization, and comprehension, in addition to basic motor skills. Clinical dementia rating (CDR) is a numeric scale measuring the severity of symptoms of dementia. The patients' cognitive and functional performances were accessed in six areas: memory, orientation, judgement and analytical, community affairs, residence and hobbies, and individual care. The patients' CDR ranks and education level are listed in Tables 2 and 3, respectively.

### 2.2. Proposed Method

**Figure 2:**
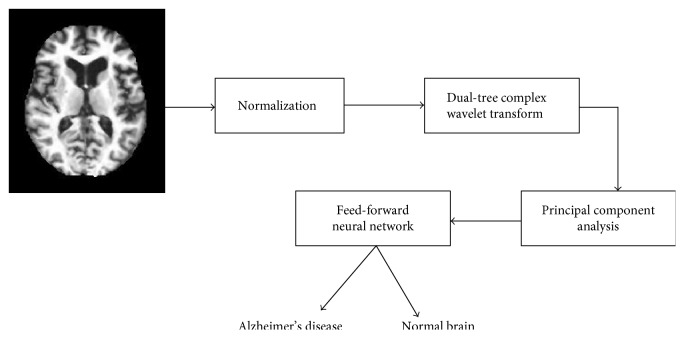
Block diagram of the proposed system.

The proposed method consists of three important stages, namely, feature extraction using DTCWT, feature dimensionality reduction using PCA, and classification using feed-forward artificial neural network. The overall block diagram of the suggested method is shown in Figure 2. Normalization of image is included in preprocessing section. All of these individual techniques have been proven outstanding, so we strongly believe that the proposed method can also achieve excellent results.

### 2.3. Image Preprocessing and Normalization

For each patient, each scanning session involves the MR of three or four T1-weighted image scans. In order to add the signal-to-noise ratio (SNR), all indicated MRI scans with the identical protocol of the same individual are motion-corrected and spatially coregistered, to the Talairach coordinate space to produce an averaged image, and then are brain-masked. The motion correction recorded the 3D images of all scans and then developed an average 3D image in initial acquisition space. Also, the scans are then resampled to 1 mm × 1 mm × 1 mm. The obtained image is converted from acquisition space to Talairach coordinate space. Lastly, the brain extraction is achieved.

We used MRIcro software (which can be downloaded from http://www.cabiatl.com/mricro/mricro/) and imported the image from the backup folder and then extracted the 2D MR image slices of each subject. In this paper, we only choose 32 important center slices from each subject manually based on our experience. These slices are used for preprocessing. The reason behind picking center slice from all slices is that it retains more relevant information about the brain tissues as compared to earlier slices and later slices in the group of 1–256 slices. The direction of the slice possibly may be sagittal, coronal, or axial. In this research, we chose axial direction by knowledge. The same process is applied to all the subjects (126 including both ADs and HCs). All images are in PNG format, and the dimensions of the slices are 176 × 208. The image is resized to 256 × 256 before being used for further processing.

### 2.4. Discrete Wavelet Transform

The discrete wavelet transform (DWT) is an image processing method [18] that gives multiscale representation of a stated signal or image [19]. Standard DWT is helpless to shift variance issue and only has horizontal and vertical directional selectivity [20]. Suppose *s* denotes a particular signal, *n* symbolizes the sampling point, *h* and *g* denote a high-pass filter and low-pass filter, respectively, and *H* and *L* depict the coefficients of high-pass and low-pass subbands. We have

(1) $$\begin{array}{c} H\left(n\right)=\underset{m}{\sum }h\left(2n-m\right)s\left(m\right),\\ L\left(n\right)=\underset{m}{\sum }g\left(2n-m\right)s\left(m\right). \end{array}$$

The *LH* represents a low-pass filter along *x*-axis and high-pass filter along *y*-axis. *HL* represents a high-pass filter along *x*-axis and low-pass filter along *y*-axis. The *LL* represents low-pass filters along both directions, and *HH* represents high-pass filters along both directions.

Here, the *HL* and *LH* have clear-cut for both vertical and horizontal orientations. For the *HH*, it combines directions of both −45 and +45 degrees jointly, which stems from the utilization of real-valued filters in DWT. This combining also hinders the direction check [21].

### 2.5. Dual-Tree Complex Wavelet Transform

The dual-tree complex wavelet transform (DTCWT) is a modified version of the traditional DWT. To help boost the directional selectivity impaired by DWT, DTCWT is proposed. The traditional DWT is shift variant because of the decimation operation used in the transform. As a consequence, a small shift in the input signal can create a very dissimilar set of wavelet coefficients formed at the output. It utilizes two real DWTs processing input data in parallel [22]. The first DWT symbolizes the real component of the transform, whereas the second DWT depicts the imaginary component together forming a complex transform.

**Figure 3:**
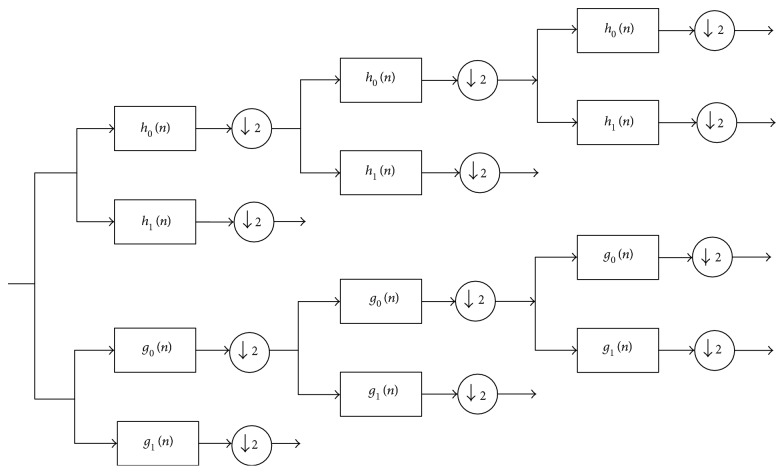
The DTCWT is implemented utilizing two wavelet filter banks functioning in parallel.

The DTCWT provides a solution for “shift-invariant problems” as well as for “directional selectivity in two or more dimensions,” which are both shortcomings of the ordinary DWT [23]. It obtains directional selectivity by utilizing wavelets that are approximately analytic. It also has the ability to produce a total of six directionally discriminating subbands oriented in the ±15, ±45, and ±75 directions, for both the real (R) and imaginary (I) parts.  Figure 3 illustrates the DTCWT. Let *h*_*i*_(*n*) and *g*_*i*_(*n*) be the filters in the first stage as in Figure 3. Let the new *k*th stage response of the first filter bank be *H*_new_^(*k*)^(*e*^*jw*^) and second filter bank be *H*_new_^′(*k*)^(*e*^*jw*^); we now have the following result as a corollary of Lemma 1.

**Corollary 1 .**

Suppose one is provided with CQF pairs {*h*_*o*_(*n*), *h*_1_(*n*)}, {*h*_*o*_′(*n*), *h*_1_′(*n*)}. For *k* > 1,

(2) $$\begin{array}{c} H_{new}^{\left(k\right)}\left(e^{jw}\right)=H\left\{H_{new}^{\prime \left(k\right)}\left(e^{jw}\right)\right\}, \end{array}$$

if and only if

(3) $$\begin{array}{c} h_{0}^{\prime \left(1\right)}\left(n\right)=h_{0}^{\left(1\right)}\left(n-1\right). \end{array}$$

A 2D image *f*(*x*, *y*) is decomposed by 2D DTCWT over a series of dilations and translations of a complicated scaling function and six complex wavelet functions *φ*_*j*,*l*_^*θ*^; that is,

(4) $$\begin{array}{c} f\left(x,y\right)=\underset{l\in Z^{2}}{\sum }s_{j_{o},l^{\phi }j_{o}},l^{\left(x,y\right)}+\underset{\theta \in \Theta }{\sum }\underset{j\geq j_{o}}{\sum }\underset{l\in z^{2}}{\sum }c_{j}^{\theta },l^{\varphi_{j}^{\theta }},l^{\left(x,y\right)}, \end{array}$$

where *θ* ∈ Θ = {±15°, ±45°, ±75°} gives the directionality of the complex wavelet function. This is to say that the decomposition of *f*(*x*, *y*) by utilizing the DTCWT creates one complex valued low-pass subband and six complex valued high-pass subbands at every level of decomposition, where every high-pass subband corresponds to one particular direction *θ*.

A study was carried out in [24] to compare the DTCWT's directional selectivity to that of the DWT. The simulation results showed that the edges detected by the DTCWT had clear contours, and nearly all directions could be detected clearly and perfectly. However, the edges detected by the DWT were discontinuous, and only horizontal and vertical edges could be successfully detected. The results verified the effectiveness of the DTCWT over the DWT. By utilizing DTCWT, we are extracting DTCWT coefficients from the preprocessed images. The additional features include the information about the demographics of the patients such as age, gender, handedness, education, SES, and clinical examination. The handedness features are not included in the work since all the patients are right-handedness.

### 2.6. Principal Component Analysis

The coefficient from the DTCWT enlarges the dimensionality of feature space that makes the classification job more complicated.

Additionally, it leads to excessive computational overhead and enormous memory storage. As a result, it is essential to lower the dimension of the feature set and get the significant features to boost the classification result. Since the last two decades, a method called PCA has earned much more attention for data visualization and reduction of dimensionality. It systematically projects the initial input data to a lower-dimensional space, well-known as principal subspace through an orthogonal transformation while preserving most of the data variations. For a stated set of likely correlated variables, these transformation outcomes in a set of values of linearly uncorrelated variables are called as principal components (PCs). All of the steps to implement PCA are demonstrated in Algorithm 1. The additional information on PCA and its implementations can be viewed in literature [25, 26].

Let us consider a set of data. PCA is employed to find a linear lower-dimensional reduction of the dataset. In this case, the variance of the constructed data is preserved. PCA limits the feature vectors to the component it selects, which leads to an effective classification algorithm. The main idea behind implementing PCA is reduction of the dimensionality of the DTCWT coefficients, which results in more adequate and accurate classification.

The following algorithm is utilized to obtain the principal components from the input matrix and finally fed to the feed-forward neural network. Now, the input matrix possesses only these PCs. Hence, the size of the matrix is reduced. Therefore, feature extraction is done in two steps: DTCWT extracts the wavelet coefficients, and essential coefficients are later selected by the PCA as described in Algorithm 1.

### 2.7. Feed-Forward Neural Networks

#### 2.7.1. Structure

**Figure 4:**
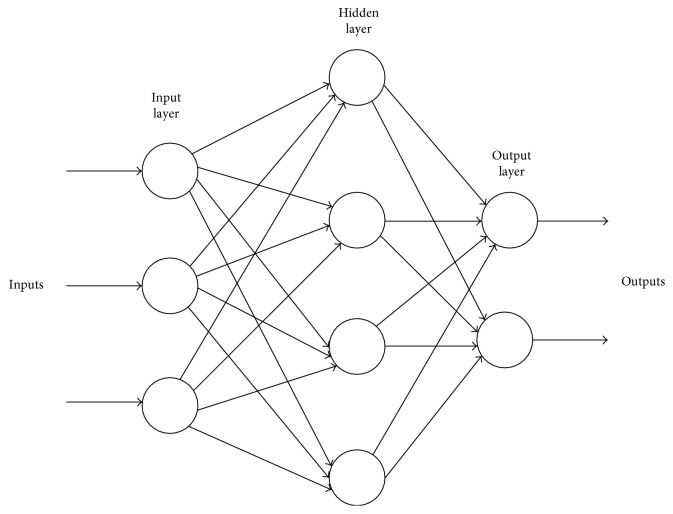
Architecture of a multilayer feed-forward neural network.

Feed-forward neural networks (FNN) are broadly used in pattern classification because they do not need any information regarding the probability distribution or a priori probabilities of distinct classes. Neural networks (NN) harness power from their densely parallel structure and their ability to acquire information from experience. As a result, they can be utilized for accurate classification of input data into different classes, provided that they are pretrained. The architecture of a multilayer feed-forward neural network is shown in Figure 4.

Three factors need to be considered in designing an ANN for a specific application: (i) the topology of the network, (ii) the training algorithm, and (iii) the neuron activation function. A network may have many layers of neurons, and its complete architecture may possess either a feed-forward or a back propagation structure. A multihidden-layer back propagation NN with sigmoid neurons in its hidden layer is chosen. Similarly, linear neurons are selected for the output layer. The training vector is provided to the NN, which is instructed batch mode [27]. The NN is a two-layer network, and its configuration is *N*_I_ × *N*_H_ × *N*_0_; *N*_I_ represents the input neurons, *N*_H_ is the hidden layer, and *N*_0_ indicates that the brain under observation is either HC or AD.

#### 2.7.2. Training Method

Mathematicians have already proven that a conjugate gradient (CG) algorithm, probing along conjugate gradient directions, produces a faster convergence than the steepest descent directions do. Among CG algorithm, the scaled conjugate gradient (SCG) method is the most powerful [28]. Thus, we utilize the SCG to train our network.

Let *ω*_1_ and *ω*_2_ be the connection weight matrix linking the input layer and hidden layer, and the hidden layer and the output layer, respectively. Later, we can deduce the training process reported by the following equations to improve these weighted values that can be divided into four subsequent steps [29].

1. The calculation of the outputs of all neurons in the hidden layer is done by

(5) $$\begin{array}{c} y_{j}=f_{H}\left(\overset{N_{I}}{\underset{i=1}{\sum }}\omega_{1}\left(i,j\right)x_{i}\right)\;j=1,2,\ldots ,N_{H}. \end{array}$$

Here, *x*_*i*_ stands for the *i*th input value, *y*_*j*_ stands for the *j*th output of the hidden layer, and *f*_H_ refers to the activation function of hidden layer, commonly a sigmoid function as observed:

(6) $$\begin{array}{c} f_{H}\left(x\right)=\frac{1}{1+\exp\left(-x\right)}. \end{array}$$

(2) The outputs of all neurons in the output layer are stated as follows:

(7) $$\begin{array}{c} \begin{array}{cc} O_{k}=f_{o}\left(\overset{N_{H}}{\underset{j=1}{\sum }}\omega_{2}\left(j,k\right)y_{j}\right) & k=1,2,\ldots ,N_{o} \end{array}. \end{array}$$

Here, *f*_o_ represents the activation function of output layer that is usually a line function. At first, all weights are accredited with random values and amended by the delta rule on the basis to the learning samples.

(3) The error is articulated as the MSE of the distinction among output and target value [30].

(8) $$\begin{array}{c} \begin{array}{cc} E_{l}=MSE\left(\overset{N_{o}}{\underset{k=1}{\sum }}O_{k}-T_{k}\right) & l=1,2,\ldots ,N_{s} \end{array}, \end{array}$$

where *T*_*k*_ depicts the *k*th value of the genuine labels which is already well-known to users and *N*_s_ denotes the number of samples [31].

(4) Let us consider that there are *N*_s_ samples; therefore, the fitness value can be written as

(9) $$\begin{array}{c} F\left(\omega \right)=\overset{N_{s}}{\underset{l=1}{\sum }}E_{l}, \end{array}$$

where *ω* designates the vectorization of the (*ω*_1_, *ω*_2_). The aim is to minimize the fitness function *F*(*w*), namely, force the output values of every sample appropriate to equivalent target values.

**Figure 5:**
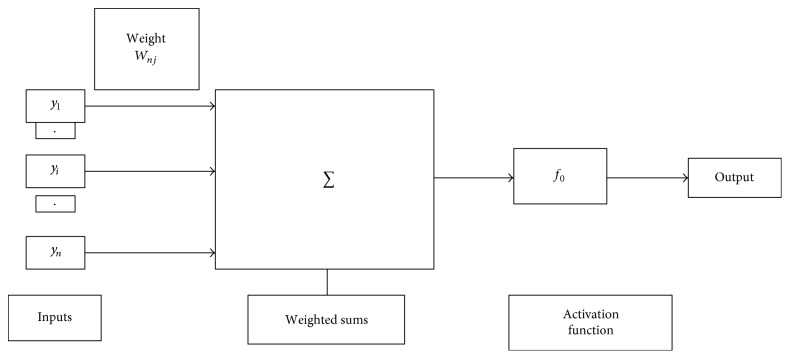
Hidden or output layer *j*. The input *j* are outputs from the previous layers. These are multiplied by their corresponding weights to configure a weighted sum. A nonlinear activation function is applied to the net input. [The inputs to input *j* are labeled *y*_1_, *y*_2_,…, *y*_*n*_. If unit *j* was in the first hidden layer, then these inputs would correspond to the input tuple *I*_1_, *I*_2_, *I*_3_,…, *I*_*n*_.]

**Figure 6:**
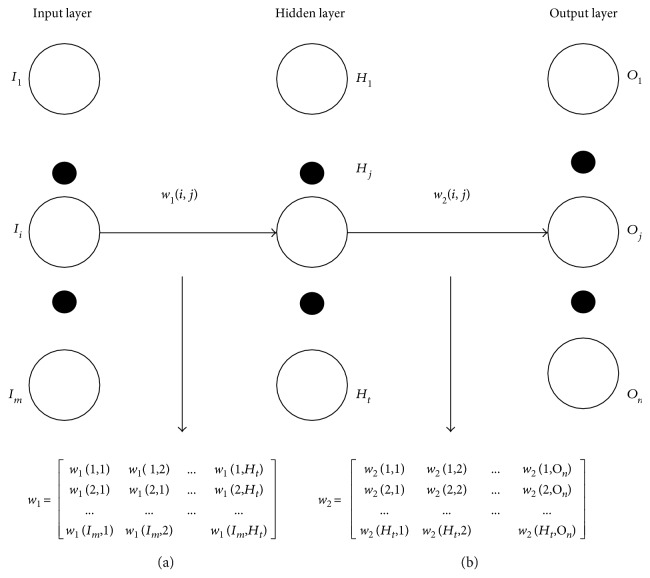
Connection weight matrix between (a) input layer and hidden layer and (b) hidden layer and output layer.

The hidden layer or the output layer *j* is depicted in Figure 5. The inputs, weighted sum, and activation function of output layer are shown in Figure 5. The connection weight between the input layer and hidden layer and hidden layer and output layer is shown in Figure 6. The connection weights can also be represented in the matrix form known as connection weight matrix.

## 3. Experiment, Results, and Discussion

The proposed method is implemented using the 32-bit Matlab 2015b environment on Intel(R) Core (TM) i3-2120, with a processing speed of 3.30 GHz and 2 GB of RAM running Microsoft Windows 7. Readers can repeat our results on any computer with which MATLAB is compatible.

This article aims at developing a CAD of AD brain system with better performance. The pseudocode is listed in Table 4.

### 3.1. Parameter Estimation for *s*

It is always a major concern to find the optimum value of decomposition level *s*. We know that a smaller *s* provides less information whereas a larger *s* provides more information to the classifier. In order to avoid overfitting problem, a smaller *s* is used. Here, we change the value of *s* from 1 to 5 with increment of 1 and check up the corresponding average accuracies with FNN. The one which gives the highest accuracy is the optimal value of *s*.

### 3.2. Feature Extraction

In this paper, we extract the DTCWT coefficients from the input images. The features of 5th resolution scales are selected because they provide higher classification performance than other resolution level scales. The DTCWT has a multiresolution representation as the wavelet transform does. For disease detection, it is preferable to use a few intermediate coefficient scales as the classifier input. The lowest scales have lost fine signal details whereas the most highly detailed scales contain mostly noise. Therefore, we prefer to choose only a few intermediate scales for the DTCWT coefficients. These obtained coefficients are sent as input to the PCA.

### 3.3. Feature Reduction

Excessive features increase calculation time as well as memory storage. In addition, they sometimes make classification much more complicated, which is known as curse of dimensionality. In this article, we utilized PCA to decrease the number of features.

Therefore, the extracted feature from DTCWT is sent to the PCA for the feature reduction. For each image, there are 768 features after 5th level of decomposition. As we have employed 32 slices for each patient, the total number of features becomes 32 × 768. Now, the image is reformed into a row vector of 1 × 24,576. The row vectors of 126 subjects are arranged into an “input matrix” with dimensions of 126 × 24,576. It is still too large for calculation. So, the input data matrix is now decomposed into the principal component “score matrix” and the “coefficient matrix.” The score matrix size after decomposition is 126 × 125. Here, the rows and columns of “score matrix” correspond to subjects and components, respectively.

**Figure 7:**
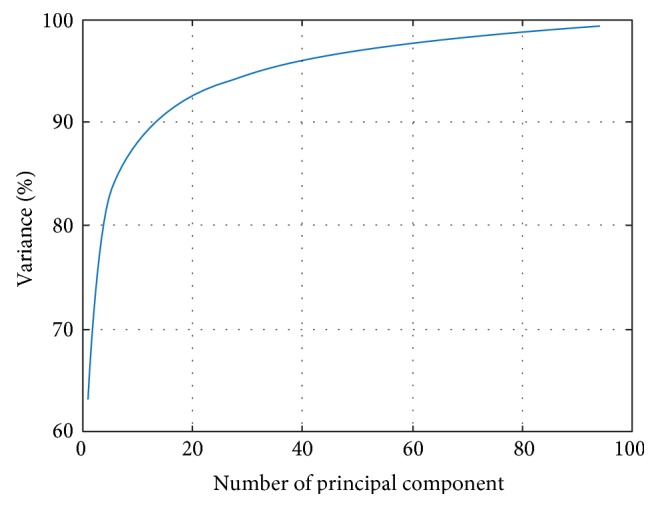
Variances versus number of principal component.

The variance with the number of principal components from 1 to 18 is listed in Table 5. Experimenting with different numbers of principal components (PCs) revealed that accuracy with PC = 14 provided the best classification accuracy preserving 90.44% of the total variance. The curve of cumulative sum of variances with the number of principal component is shown in Figure 7. We did not set the energy threshold as 95% because that would cost too many features, along with computational burden.

### 3.4. BPNN Training

The 14 PCs are directly sent to BPNN. Thus, the number of input neurons *N*_*I*_ is 14. Then, the number of hidden layer neurons (*N*_*H*_) is determined as 10 according to the information entropy method [32]. Therefore, the architecture of the neural network becomes 14-10-1. The SCG method is employed because it is extremely faster than BP, MBP, and ABP [28].

### 3.5. Performance Measures

There are several techniques to evaluate the efficiency of classifiers. The performance is calculated on the essence of the overall confusion matrix. It holds the correct and incorrect classification results.  Table 6 shows a confusion matrix for binary classification, where TP, TN, FP, and FN depict true positive, true negative, false positive, and false negative, respectively, as illustrated in Table 7.

Here, AD brains are assumed to hold the value “true” and NC ones are assumed to hold the value “false” following normal convention.

The accuracy is the most accepted empirical measure to access effectiveness of classifier. It is formulated by

(10) $$\begin{array}{c} Accuracy=\frac{TP+TN}{TP+TN+FP+FN}. \end{array}$$

Sensitivity is the measure of the proportion of true positives that are correctly classified, and specificity is the measure of the proportion of negatives which are correctly classified. These are calculated by

(11) $$\begin{array}{c} Sensitivity=\frac{TP}{TP+FN},\\ Specificity=\frac{TN}{TN+FP}. \end{array}$$

The precision and the recall are formulated by

(12) $$\begin{array}{c} Precision=\frac{TP}{TP+FP},\\ Recall=\frac{TP}{TP+FN}. \end{array}$$

### 3.6. Statistical Analysis

In order to execute a strict statistical analysis, stratified cross-validation (SCV) is used. We apply a 10-fold CV technique in this experiment because of two reasons: (1) to make balance between reliable estimate and computational cost and (2) for providing a fair comparison because the common convention was to take the value of *K* equal to 10 [33].

A 10-fold CV means we have to divide our dataset randomly into ten mutually exclusively folds of approximately equal size and almost the same distribution. In each run, 9 subsets will be used for training, and the remaining one will be utilized for the validation. This process is repeated 10 times, in which every subset is utilized for validation once. The 10-fold CV is repeated 50 times; namely, a 50x 10-fold CV is implemented.

The accuracies, sensitivities, and specificities obtained from the 50 runs of 10-fold CV are presented in Table 8. Our method achieved an accuracy of 90.06 ± 0.01%, a sensitivity of 92.00 ± 0.04%, a specificity of 87.78 ± 0.04%, and a precision of 89.6 ± 0.03%.

### 3.7. Comparison to Other State-of-the-Art Approaches

To further determine the effectiveness of the proposed “DTCWT + PCA + FNN,” we compared it with seven state-of-the-art approaches in Table 8. Some of these approaches utilized different statistical settings, making direct comparison difficult. The results in Table 8 show that study [34–37] did not present standard deviations (SD) of three standards. The specificities of study [34–36] are lower than those demonstrated by other methods. Therefore, these three methods are not worthy for further study. Similarly, study [37] obtained a classification specificity of 100%. In spite of its high specificity, both the accuracy and sensitivity achieved by this algorithm are poor. Hence, this method is also not considered further for the study. Three other methods reported both mean values and standard deviation values. They also achieved satisfying results. Study [38] obtained promising results because of the voxel-based morphometry (VBM). Indeed, VBM has frequently been employed to study brain changes. Study [37] demonstrated that a taxi driver will normally have a larger back section of the posterior hippocampus. Study [39] concluded that global gray matter decreases linearly with old age but global white matter remains in the same amount. Nevertheless, VBM requires an accurate spatial normalization, or the classification accuracy may decrease significantly. Study [40] was based on a novel approach called the displacement field (DF). This study measured and estimated the displacement field of various slices between AD and HC subjects. There are other methods that have distinguished AD from HC; however, they dealt with images formed by other modalities: PET, SPECT, DTI, and so forth. Hence, they are also not considered in this study.

**Figure 8:**
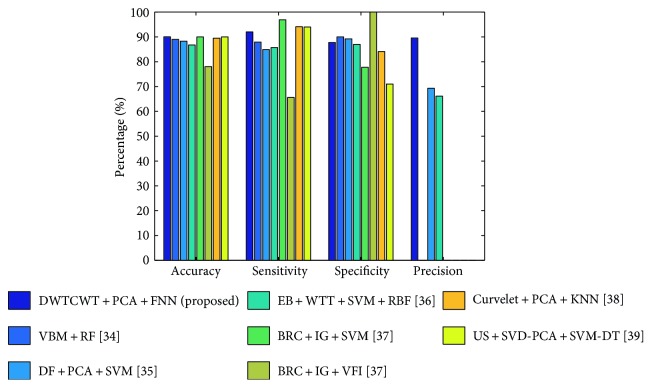
Bar plot of the algorithm comparison ([34–36, 38] did not mention its precision).

Finally, the proposed “DTCWT + PCA + FNN” achieved an accuracy of 90.06 ± 0.01%, a sensitivity of 92.00 ± 0.04%, a specificity of 87.78 ± 0.04%, and a precision of 89.60 ± 0.03%. With respect to classification accuracy, our approach outperforms five other methods and is almost equal to the accuracies of the remaining two methods that did not account for means and standard deviations. We also achieved a promising sensitivity and a promising specificity. Hence, our results are either better than or comparable to those of the other methods. The bar plot of the algorithm comparison is shown in Figure 8. The acronyms list is depicted in Table 9.

## 4. Conclusions and Future Research

We presented an automated and accurate method for AD identification based on a DTCWT, PCA, and FNN. The results showed that the proposed method achieved an accuracy of 90.06 ± 0.01%, a sensitivity of 92.00 ± 0.04%, a specificity of 87.78 ± 0.04%, and a precision of 89.6 ± 0.03% and outperformed 7 state-of-the-art algorithms.

We will focus our future research on the following aspects: (i) testing other advanced variants of wavelet such as 3D-DTCWT, wavelet packet analysis, and fractional calculus; (ii) utilizing different feature reduction techniques such as independent component analysis (ICA) [41], linear discriminant analysis (LDA) [42], probabilistic PCA [43], or sparse-autoencoders [44]; (iii) testing our data with least-square techniques [45], kernel support vector machine (k-SVM), such as fuzzy SVM [46], radial basis function neural network (RBFNN) [47], deep learning methods such as convolutional neural network (CNN) [48], and other alternative pattern recognition tool for classification; (iv) utilizing advanced swarm intelligence techniques such as particle swarm optimization [49], artificial bee colony [50], genetic pattern search [51], ant colony optimization [52], and biogeography-based optimization [53] to find the optimal kernel; (v) testing the proposed method on images obtained from different modalities such as computed tomography (CT) [54], ultrasound, spectrum imaging [55], and 3D MRI; (vi) utilizing other advance image preprocessing technique to enhance the classification performance, such as image denoising, image enhancement, and image segmentation; and (vii) classification may be carried out on the sparsity domain.

## Figures and Tables

**Algorithm 1:**
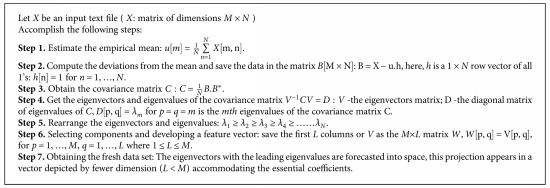
PCA algorithm.

**Table 1:** Statistical data of the participants.

Factor	HC	AD
No. of patients	98	28
Age (years)	75.91 ± 8.98	77.75 ± 6.99
Education	3.26 ± 1.31	2.57 ± 1.31
Socioeconomic status	2.51 ± 1.09	2.87 ± 1.29
CDR	0	1
MMSE score	28.95 ± 1.20	21.67 ± 3.75
Gender (M/F)	26/72	9/19

**Table 2:** Clinical dementia rating scale.

CDR	Rank
0	Nondementia
0.5	Very mild dementia
1	Mild dementia
2	Moderate dementia

**Table 3:** Education codes.

Code	Description
1	Beneath high school graduate
2	Secondary school graduate
3	Some college
4	College graduate
5	Above college

**Table 4:** Pseudocode of the proposed system.

Step 1: Import.
(a) Import the OASIS dataset.
(b) Ensure MRI as normal or abnormal brain.
Step 2: Resample the image into 256 × 256.
Step 3: Compute 5-level DTCWT on the preprocessed images.
Step 4: Perform PCA on the obtained matrix. The selected number of principal component (PC) should preserve at least 90% of total variances.
Step 5: Train feed-forward neural network by taking input as reduced set of feature vectors and their corresponding class labels.
Step 6: Evaluation
(a) Obtain the confusion matrix.
(b) Calculate the classification accuracy and other essential parameters.

**Table 5:** Detailed data of PCA.

No. of prin. comp.	1	2	3	4	5	6	7	8	9
Variance (%)	63.18	72.15	77.08	80.28	83.05	84.55	85.68	86.59	87.47
No. of prin. comp.	10	11	12	13	14	15	16	17	18
Variance (%)	88.18	88.28	89.41	89.96	90.44	90.86	91.23	91.58	91.91

**Table 6:** Confusion matrix for a binary classifier to discriminate between two classes (*A*_1_ and *A*_2_).

True class	Predicted class	
	*A*_1_ (patients)	*A*_2_ (controls)
*A*_1_ (patients)	TP	FN
*A*_2_ (controls)	FP	TN

**Table 7:** Evaluation indicators.

Indicator	Explanation
TP	True positive, anticipating an AD to AD
FP	False positive, anticipating an HC to AD
TN	True negative, anticipating an HC to HC
FN	False negative, anticipating an AD to HC

**Table 8:** Algorithm performance comparison for MRI brain image.

Algorithm	Accuracy (%)	Sensitivity (%)	Specificity (%)	Precision (%)
DTCWT + PCA + FNN (proposed)	**90.06 ± 0.01**	**92.00 ± 0.04**	**87.78 ± 0.04**	**89.6 ± 0.03**
VBM + RF [38]	89.0 ± 0.7	87.9 ± 1.2	90.0 ± 1.1	N/A
DF + PCA + SVM [40]	88.27 ± 1.9	84.93 ± 1.21	89.21 ± 1.6	69.30 ± 1.91
EB + WTT + SVM + RBF [56]	86.71 ± 1.93	85.71 ± 1.91	86.99 ± 2.30	66.12 ± 4.16
BRC + IG + SVM [34]	90.00	96.88	77.78	N/A
BRC + IG + VFI [34]	78.00	65.63	100.00	N/A
Curvelet + PCA + KNN [35]	89.47	94.12	84.09	N/A
US + SVD-PCA + SVM-DT [36]	90.00	94.00	71.00	N/A

**Table 9:** Acronyms list.

Acronym	Definition
AD	Alzheimer's disease
HC	Healthy control
MR(I)	Magnetic resonance (imaging)
DTCWT	Dual-tree complex wavelet transform
PCA	Principal component analysis
FNN	Feed-forward neural network
DWT	Discrete wavelet transform
OASIS	Open Access Series of Imaging Studies
MMSE	Mini mental state examination
CDR	Clinical dementia rating
SNR	Signal-to-noise ratio
VBM	Voxel-based morphometry
RF	Random forest
DF	Displacement field
SVM	Support vector machine
EB	Eigenbrain
WTT	Welch's *t*-test
RBF	Radial basis function
BRC	Brain region cluster
IG	Information gain
KNN	*k*-nearest neighbor
ANN	Artificial neural network
SCV	Stratified cross-validation
